# Hypoglycemia and hyperglycemia in extremely low-birth-weight infants

**DOI:** 10.1186/1824-7288-41-S1-A7

**Published:** 2015-09-24

**Authors:** Maria Pia De Carolis, Serena A Rubortone, Carmen Cocca, Giovanni Pinna, Eloisa Tiberi, Zecca Enrico, Costantino Romagnoli, Silvia Salvi, Sara De Carolis

**Affiliations:** 1 Division of Neonatology, Department of Obstetrics, Gynecology and Paediatrics, Catholic University of the Sacred Heart, Rome, 00168, Italy; 2 High Risk Pregnancies Division, Department of Obstetrics, Gynecology and Paediatrics, Catholic University of Sacred Heart, Rome, 00168, Italy

## Background

Glucose metabolism disorders are common in extremely low birth weight (ELBW) infants and are associated with high morbidity and mortality [1-9]. This study was conducted to evaluate the prevalence and risk factors associated with both hypo and hyperglycemia in ELBW infants.

## Materials and methods

All inborn ELBW neonates admitted to our NICU during a 5-year period were eligible for this retrospective analysis. Exclusion criteria were: birth weight (BW) <400 grams, major congenital malformations, death during the first 24 hours of life. Hypoglycemia was defined as blood glucose level (BGL) ≤45 mg/dL; hyperglycemia as BGL>240 mg/dL in a single determination or >180 mg/dL in two determinations at 2-hour intervals. Continuous intravenous insulin infusion was started after an ineffective glucose restriction.

## Results

Of 195 ELBW infants, 29 (14.8%) were excluded and 166 (GA 26.7 2.1 weeks, BW 751 152 grams) were analyzed and grouped to their BGL. Normoglycemia was observed in 79 neonates (47.6%) (N-Group); 80 neonates (52.4%) showed abnormal BGL: 21 (12.7%) were hypoglycemic (Hypo-Group), 53 (31.9%) hyperglycemic (Hyper-Group) and 13 (7.8%) showed both hypoglycemia and hyperglycemia (Hypo&Hyper-Group). Clinical characteristics of the groups are reported in Table 1. Hypo-Group respect to N-Group showed a higher rate of small for gestational age (SGA) neonates (p=0.03). Hyper-Group in comparison to N-Group showed a tendency toward a lower GA (p=0.05), lower BW (p<0.001), higher sepsis rate (p<0.001), higher rate of treatment with inotropic agents (p=0.02), corticosteroids (p=0.006) and nonsteroidal antiinflammatory drugs (p=0.01). Hypo&Hyper-Group respect to N-Group showed similar GA, lower BW (p<0.001), higher sepsis rate (p<0.01), higher rate of inotropic treatment (p=0.04). Insulin was administered in 35 neonates (66%) of Hyper-Group and in 8 neonates (61.5%) of Hypo&Hyper-Group. Intraventricular Hemorrhage** (** IVH) rate was higher in Hyper-Group and Hypo&Hyper-Group respect to N-Group (p=0.002) as well as IVH grade3 (p=0.001 and p=0.02, respectively).The rate of both Retinopathy of Prematurity** (** ROP) and ROP ≥stage 2 in survived neonates was higher in Hyper-Group respect to N-Group (p=0.008 and p=0.002, respectively). Mortality was similar among the groups (Table 2).

**Table 1:** Demographic data and risk factors in the study groups

	N-Group N=79	Hypo-Group N=21	Hyper-Group N=53	Hypo&Hyper-Group N=13
GA (wks), *mean±SD*	26.8±2.0	27.7±2.4	26.1±2.1	26.8±1.8
BW (g), *mean±SD*	808±136	719±140	695±146	692±140
Male, n (%)	17 (21.5)	10 (47.6)	26 (49.0)	8 (61.5)
SGA, n (%)	22 (27.8)	11 (52.3)	16 (30.1)	4 (30.7)
Apgar^1^ <6, n (%)	39 (49.3)	9 (42.8)	34 (64.1)	8 (61.5)
Apgar^5^ <6, n (%)	8 (10.1)	0	9 (16.9)	2 (15.3)
Intubation, n (%)	47 (59.4)	11 (52.3)	37 (69.8)	10 (76.9)
Antenatal Steroid, n (%)	64 (81.0)	17 (80.9)	41 (77.3)	12 (92.3)
RDS, n (%)	66 (83.5)	19 (90.4)	49 (92.4)	13 (100)
Sepsis, n (%)	16 (20.2)	4 (19.0)	32 (60.3)	7 (53.8)
Inotropic Agents, n (%)	26 (32.9)	6 (28.5)	28 (52.8)	8 (61.5)
Xanthines, n (%)	70 (88.6)	20 (95.2)	50 (94.3)	13 (100)
Postnatal Steroid, n (%)	11 (13.9)	3 (14.2)	18 (33.9)	3 (23.0)
NEC, n (%)	6 (7.5)	2 (9.5)	5 (9.4)	4 (30.7)
Medical treatment for PDA, n (%)	34 (43.0)	8 (38.0)	34 (64.1)	9 (69.2)
Surgical Procedures, n (%)	5 (6.3)	3 (14.2)	7 (13.2)	2 (3.7)

RDS: Respiratory Distress Syndrome; PDA: Patent Ductus Arteriosus; NEC: Necrotizing enterocolitis

**Table 2:** Complications and outcome in the study groups

	N-Group N=79	Hypo-Group N=21	Hyper-Group N=53	Hypo&Hyper-Group N=13
IVH, n (%)	17 (21.5)	7 (33.3)	25 (47.1)	8 (61.5)
IVH (grade 3, n (%)	5 (6.3)	1 (4.7)	15 (28.3)	4 (30.7)
ROP all stages in the survivors, n (%)	49 of 61 (80.3)	10 of 16 (62.5)	35 of 35 (100)	7 of 8 (87.5)
ROP > stage 2 in the survivors, n (%)	35 of 71 (57.3)	9 of 16 (56.2)	35 of 35 (100)	6 of 8 (75)
Mortality, n (%)	18 (22.7)	5 (23.8)	18 (33.9)	6 (46.1)

IVH: IntraventricularHemorrhage; ROP: Retinopathy of Prematurity

## Conclusions

Among ELBW infants, hypoglycemia occurs more frequently in SGA neonates, while hyperglycemia alone or a marked variability of BGL (hypo and hyperglycaemia) is more common in sick neonates. High rate of glucose homeostasis disorders highlights the importance of carefully monitoring BGL in order to a prompt management. Continuous glucose monitoring recently used in neonates [10] might be a useful tool for monitoring glucose changes also in ELBW neonates.
